# Jack stone in the bladder: case report of a rare entity

**DOI:** 10.1186/s12894-017-0230-6

**Published:** 2017-06-05

**Authors:** Duminda Subasinghe, Serozsha Goonewardena, Vickneswaran Kathiragamathamby

**Affiliations:** Department of Urology, The National Hospital of Sri Lanka, Colombo, Sri Lanka

**Keywords:** Cystolitholapaxy, Jack stones,Transurethral resection, Urinary bladder

## Abstract

**Background:**

Jackstone is a bladder stone that has a similar appearance to toy jacks. However review of the English language medical literature revealed only a few previous reports of jackstone calculus.

**Case presentation:**

We report a case in which a large jackstone calculus was incidentally detected during the evaluation of 67 year old male presenting with lower urinary tract symptoms. X-Ray kidney, ureter, bladder showed a large irregular shaped radio-opaque shadow in the pelvic region. He underwent cystolitholapaxy and transurethral resection of the prostate.

**Conclusion:**

It is important to recognize the characteristic shape of the urinary bladder calculi in the diagnosis of the jack stones and to treat the primary cause of calculi formation.

## Background

This calculi type is referred as a “jack” stone because of its similar appearance to the children’s game toys, Jacks. The commonest site of Jackstones is urinary bladder and rarely in the upper urinary tract. They are almost always composed of calcium oxalate dihydrate. They grow forming radiating spicules due deposition of new minerals resulting in irregular shape. Here we describe a patient that presented with obstructive lower urinary tract symptoms and Jack stones in the urinary bladder.

## Case presentation

A 67-year-old male presented with worsening, predominantly voiding lower urinary tract symptoms of 4 years duration with worsening of symptoms for last 1 year duration. He had hesitancy, poor stream of urine, frequency of micturition.Also he had experienced only one episode of visible, painful haematuria without clots at the end of the urinary stream which settled with one week treatment with ayurvedic medications. There was no history of other symptoms such as dysuria, fever suggestive of urinary sepsis, acute or chronic retention of urine, urgency, nocturia, nocturnal enuresis or episodes of urinary incontinence or passage of calculi. There was no history of previous urinary tract surgeries or invasive procedures such as catheterization. His past medical history was unremarkable. The physical examination was unremarkable except clinically benign prostatomegaly (about 40 g).

**Fig. 1:**
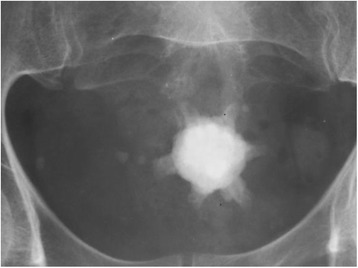
XRay KUB showing a irregular shaped stone

**Fig. 2:**
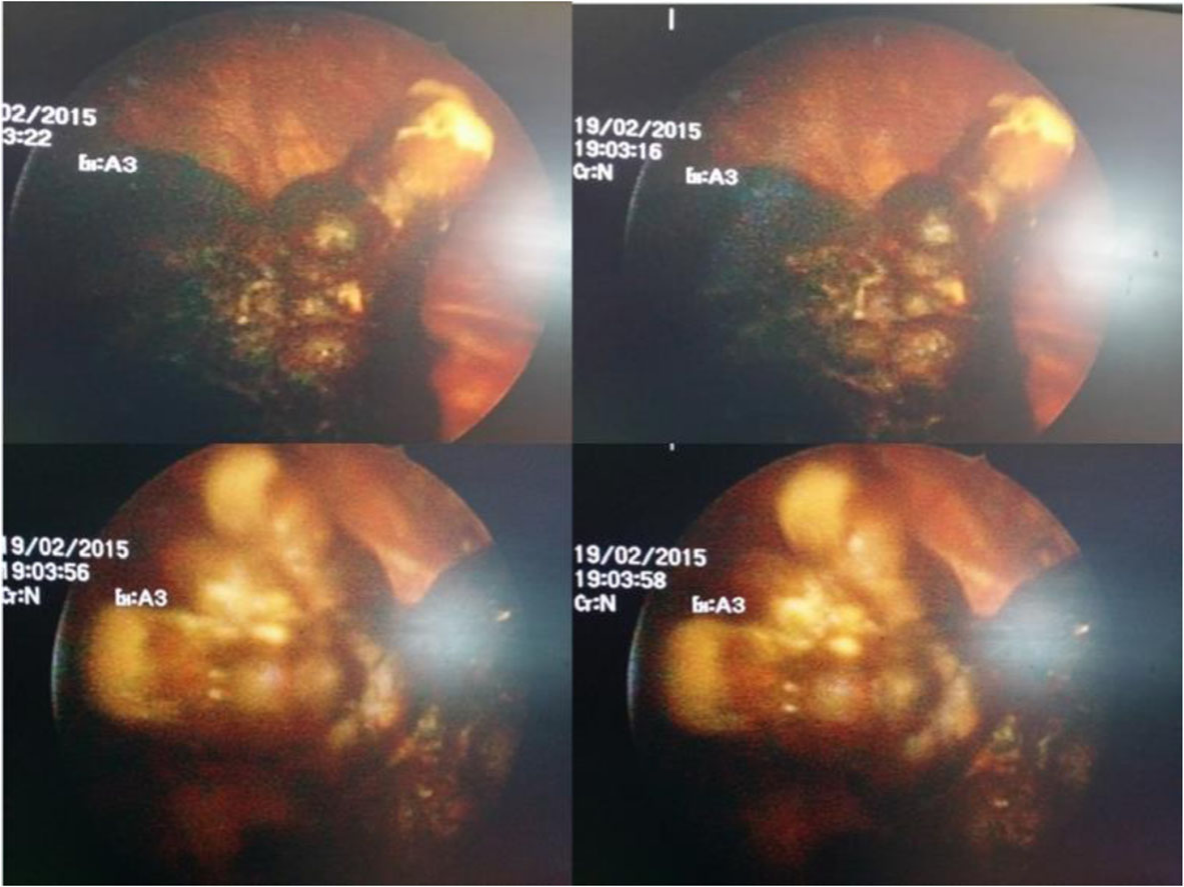
Cystoscopic view of intravesicle Jack stone

**Fig. 3:**
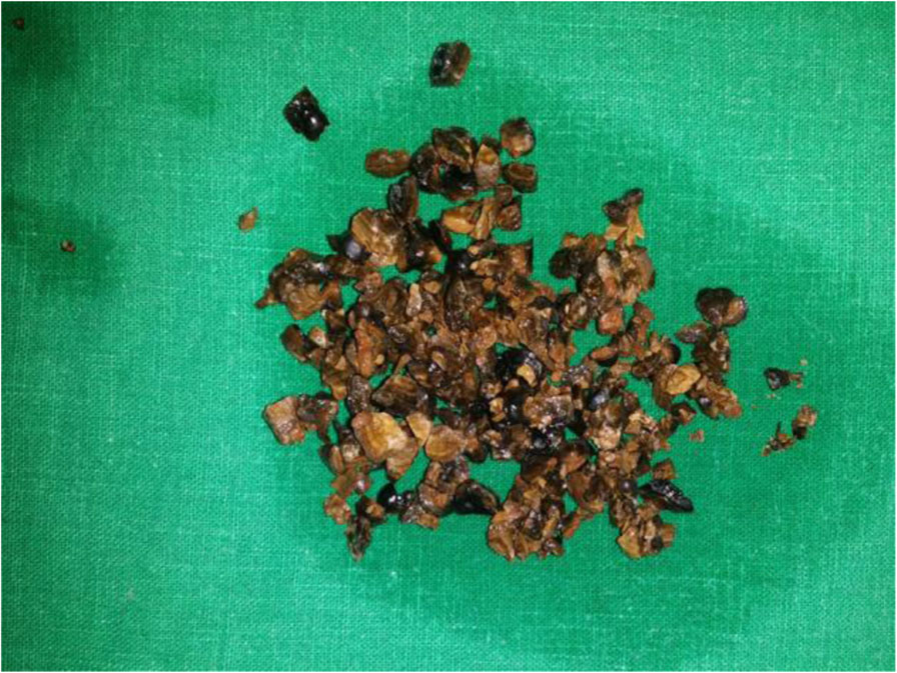
Fragmented Jack stone following cystoscopic litholapaxy

His uroflowmetry showed a significant limitation of maximum flow rate (Q max = 4.2 ml/s). On ultrasonography he was found to have 50 g prostate with slightly thickened bladder wall (5 mm) with a large stone (2.5x3cm) in the urinary bladder. He had a significant post voidal volume (109 ml) in the presence of 376 ml of prevoidal volume of urine. Upper parts of the urinary tracts were sonogrphically normal. X-Ray KUB showed a large irregular shaped radio-opaque shadow in the pelvic region [Fig. 1]. Hematological, biochemistry investigations did not reveal any abnormality. Patient underwent cystoscopy which revealed a large Jack stone in urinary bladder [Fig. 2].Cystolitholopaxy was performed and multiple small stones (Fig. 3) were retrieved. He also underwent transurethral resection of the prostate which revealed 50 g prostate. The operation time was 60 min. His post-operative period was uneventful and he was started on alpha-1A blockers in the postoperative period and Foley’s catheter was removed on the 4th postoperative day. At follow-up of three weeks after the surgery the patient was voiding with good stream.

## Discussion

Only few cases of jackstones had been reported previously [1, 2]. As the name implies this variety of stone has a characteristic shape resembling a child’s toy. These types of stone are commonly described in the veterinary literature with common occurrence in cattle, cats and dogs. Dogs are mostly commonly affected and are usually composed of silica [3]. Calcium oxalate is the most common component of urinary calculi [4]. Jackstone calculi have a characteristic shape that suggests the specific mineral content of these stones. This can have therapeutic implications. Although this typical shape has been reported with the use of other imaging modalities in human patients and has also been reported on sonograms in various animal species. Dihydrate stones tend to be fragmented by lithotripsy more easily than monohydrate stones. Jackstone calculi in humans are usually specific for calcium oxalate dihydrate stones [5].

Bladder outlet obstruction remains the most common cause of bladder calculi in adults. Most common factors predisposing to bladder stone formation includes prostatic disease, previous lower urinary tract surgery, metabolic abnormalities, intravesical foreign bodies, spinal cord injuries, upper urinary tract calculi [6]. Stones forming due to the above mentioned factors are usually not jackstones. The presentation of vesical calculi varies from completely asymptomatic to symptoms of supra pubic pain, dysuria, hesitancy, intermittency, nocturia, frequency and urinary retention. In our patient the bladder out flow obstruction due to benign prostatic enlargement is the likely cause of this stone. Enlarged prostate probably restricts the calculus into its eccentric location and contributes to the growth of stone by causing stasis of urine.

It is important to recognize the characteristic shape of the jackstones as they are susceptible to lithotripsy. We were able to manage his problems, vesicle stone and bladder out flow obstruction by minimally invasive surgery in single index admission.

## Conclusion

It is important to recognize the characteristic shape of the urinary bladder calculi on radiological investigation in the diagnosis of the jack stones. Identification and treatment of the primary cause of calculi formation is important in the overall management.

## Abbreviation

*KUB*: kidney, ureter, bladder

## References

[1] Singh KJ, Tiwari A, Goyal A (2011). Jackstone: a rare entity of vesical calculus. Indian J Urol.

[2] Perlmutter S, Hsu CT, Villa PA, Katz DS (2002). Sonography of a human jackstone calculus. J Ultrasound Med.

[3] Osborne CA, Clinton CW, Kim KM, Mansfield CF (1986). Etiopathogenesis, clinical manifestations and management of canine silica urolithiasis. Vet Clin North America Small Anim Pract.

[4] Prien EL., Sr. The analysis of urinary calculi. Urol Clinic North Am 1974;1:229–240. 4610942

[5] Dretler S (1988). Stone fragility: a therapeutic distinction. J Urol.

[6] Douenias R, Rich M, Badlani G, Mazor D, Smith A (1991). Predisposing factors in bladder calculi.Review of 100 cases. Urology.

